# Phillyrin restores metabolic disorders in mice fed with high-fat diet through inhibition of interleukin-6-mediated basal lipolysis

**DOI:** 10.3389/fnut.2022.956218

**Published:** 2022-10-05

**Authors:** Zhizheng Fang, Lu Wei, Yanping Lv, Tongsheng Wang, Hamizah Shahirah Hamezah, Rongchun Han, Xiaohui Tong

**Affiliations:** ^1^School of Pharmacy, Anhui University of Chinese Medicine, Hefei, China; ^2^School of Life Sciences, Hainan University, Haikou, China; ^3^School of Life Sciences, Anhui University of Chinese Medicine, Hefei, China; ^4^Institute of Systems Biology, Universiti Kebangsaan Malaysia (UKM), Bangi, Malaysia

**Keywords:** interleukin-6, lipolysis, obesity, adipose triglyceride lipase, phillyrin

## Abstract

The function of white adipose tissue as an energy reservoir is impaired in obesity, leading to lipid spillover and ectopic lipid deposition. Adipose tissue inflammation can reduce the efficacy of lipid storage in adipocytes by augmenting basal lipolysis through producing interleukin-6 (IL-6). Therefore, pharmacological compounds targeting adipose tissue inflammation or IL-6 signaling might have the potential to combat obesity. This study aims to investigate the impact of Phillyrin, which is frequently used for treating respiratory infections in clinics in China, on obesity-related metabolic dysfunctions. Firstly, a mouse model of diet-induced obesity is used to assess the pharmacological applications of Phillyrin on obesity *in vivo*. Secondly, *ex vivo* culture of adipose tissue explants is utilized to investigate actions of Phillyrin on IL-6-linked basal lipolysis. Thirdly, a mouse model of IL-6 injection into visceral adipose tissue is explored to confirm the anti-basal lipolytic effect of Phillyrin against IL-6 *in vivo*. The results show that Phillyrin treatment reduces circulating level of glycerol, decreases hepatic steatosis and improves insulin sensitivity in obese mice. Meanwhile, Phillyrin attenuates obesity-related inflammation and IL-6 production in adipose tissue in obese mice. Furthermore, Phillyrin treatment results in resistance to IL-6-induced basal lipolysis in adipose tissue through suppressing expression of adipose triglyceride lipase (ATGL) both *in vivo* and *in vitro*. Collectively, these findings suggest that Phillyrin can restrain lipid efflux from inflamed adipose tissue in obesity by inhibiting IL-6-initiated basal lipolysis and ATGL expression, and thus is a potential candidate in the treatment of obesity-associated complications.

## Introduction

Dysfunctions of white adipose tissue (WAT) are the center of diverse adverse outcomes associated with obesity (1). Unrestrained basal lipolysis in WAT occurs in obesity and results in ectopic lipid accumulation in liver, skeletal muscles or cardiovascular system, contributing to obesity-associated metabolic derangements (2, 3). Arner et al. report that high spontaneous (basal) lipolysis in subcutaneous fat is linked to weight gain and disturbed glucose metabolism in women (4). Normally, mobilization of fatty acids in adipocytes is exquisitely regulated, displaying both spontaneous (basal) and hormone-stimulated lipolysis (5, 6). The sequential hydrolysis of triglycerides to produce glycerol and free fatty acids (FFA) in lipid droplets in adipocytes, requires at least three distinct hydrolases: Adipose triglyceride lipase (ATGL, also annotated as PNPLA2), hormone-sensitive lipase (HSL) and monoglyceride lipase (MGL), which consecutively release three molecules of FFA from the glycerol backbone (7). ATGL, catalyzing the first step of lipolysis and converting triglyceride to diacylglycerol and FFA, is considered the main enzyme responsible for lipolysis (8, 9). Recently, pharmacological inhibition of ATGL to restrain basal lipolysis in adipose tissue has been demonstrated to be a potentially powerful therapeutic strategy to combat obesity and associated metabolic disorders (10, 11).

Obesity-related adipose tissue inflammation plays an important role in accelerating basal lipolysis in adipocytes via secreting cytokines (2, 12, 13). Remarkably, overproduced interleukin-6 (IL-6) from adipose tissue macrophages (ATM) in obesity accounts for elevated basal lipolysis, which leads to production of excessive acetyl-CoA in the liver eliciting hepatic glucose production (HGP) and insulin insensitivity in mice (2, 14). Thus, small molecules targeting inflammation or IL-6 in adipose tissue might be helpful in limiting excessive basal lipolysis and lipid breakdown in adipose tissue in obesity.

Phillyrin, one of the main active ingredients from *Forsythia suspensa* (Thunb.) Vahl (Oleaceae), is frequently used for treatments of respiratory infectious diseases in clinics in China. Phillyrin has been reported to exhibit anti-inflammatory effects against lipopolysaccharide (LPS)-induced osteolysis (15), acute kidney injury (16) and pulmonary inflammation (17) in mice, and traumatic brain injury in mouse model (18). But whether this drug has any implication in adipose tissue inflammation in obesity hasn’t been examined. Interestingly, this drug is also shown to inhibit weight gain in diet-induced obesity in mice (19). Thus, we wonder whether Phillyrin can be applied to inhibit obesity-related adipose inflammation and metabolic syndrome. To address these questions, we mainly evaluate effects of Phillyrin treatment in high-fat diet (HFD)-fed mice. We show that Phillyrin treatment retards HFD-fed induced weight gain, reduces plasma levels of glycerol, and hepatic steatosis in mice in relation to its suppression of adipose tissue inflammation and IL-6 production. Moreover, Phillyrin treatment reverses augmented basal lipolysis in adipose tissue by IL-6 associated with ATGL inhibition both *in vitro* and *in vivo*. Taken together, these data reveal an essential role for Phillyrin in restoring IL-6-linked excessive basal lipolysis and indicate a potential for targeting IL-6/ATGL signaling to combat metabolically unhealthy obesity.

## Materials and methods

### Animals and animal care

Male C57BL/6J mice were obtained from GemPharmatech (Nanjing, China) at ages ranging from 4 to 6 weeks old. Mice were housed in a pathogen-free facility, with free access to autoclaved water and were maintained on a 12-h light/dark cycle. Body weight was recorded weekly. All mice were fed normal chow diet unless otherwise indicated. For high-fat feeding, a 60% (by calories) fat diet (XTHF60, irradiated; Xietong Shengwu, Nanjing, China) was used. After 1 week of accommodation, mice were administrated with corresponding drugs. Metformin (1 mg/kilogram body weight per day, i.g.) was used as positive control. Phillyrin was gavaged at the concentrations of 25 and 50 mg/kilogram body weight daily. Control mice were gavaged with vehicle (0.1% w/v carboxymethylcellulose sodium). Phillyrin and Metformin were purchased from Sigma-Aldrich (Shanghai, China). Wildtype male C57BL/6J mice fed on normal diet were used for the experiments of IL-6 (Sangon Biotech, Shanghai, China) injection into perigonadal white adipose tissue (gWAT). 1 μg of IL-6 dissolved in 50 μl PBS was injected into per depot of gWAT per mouse, after the mice were anesthetized with Zoletil 50 (50 mg/kg, Virbac, France) and the abdomen was surgically opened. All animal procedures were approved by the Institutional Animal Care and Use Committee (IACUC) at Anhui University of Chinese Medicine.

### Tolerance tests

Tolerance tests were monitored in mice that were fasted for 16 h. For oral glucose tolerance test (OGTT), mice received an i.g. gavage of 2 g per kilogram of body weight. For the insulin tolerance test (ITT), mice received an i.p. injection of 0.5 IU porcine insulin (MACKLIN, Shanghai, China) per kilogram of body weight. For the pyruvate tolerance test (PTT), pyruvate (Sangon Biotech, Shanghai, China) at a dose of 2 g/kg body weight was injected intraperitoneally to mice after fasting. Blood glucose levels at indicated hours were measured by a glucometer (Accu-Chek, Roche, China).

### Food intake measurement

For food intake studies, mice were acclimated to custom-made food racks for a week prior to measurements. Mice were provided with fresh cages to avoid the leftover of food spilling in the bedding. Food intake was measured by weighing the food racks containing food pellets before and after 24-h, which was continued for 7 continuous days.

### Histological analysis and oil red O staining

Liver and adipose tissue were immediately excised and parts were fixed in 4% paraformaldehyde after mice were sacrificed and then embedded in paraffin. Sections of 5 μm were stained with hematoxylin and eosin (HE) (Solarbio, China). Other parts of liver were snap frozen with liquid nitrogen and then cryostat-sectioned at a thickness of 10 μm onto glass slides for oil red O staining. These sections were fixed with 4% paraformaldehyde and briefly washed with running tap water. Then, sections were rinsed with 60% isopropanol for 1 min, followed by staining with freshly prepared oil red O working solution (Solarbio, China) for 15 min, and differentiated with 60% isopropanol for 1 min. Nuclei were briefly stained with Mayer’s Hematoxylin solution (Solarbio, China), followed by rinsing with distilled water. Sections were mounted with glycerol gelatin aqueous slide mounting medium (Solarbio, China). Positive staining was quantified using Image J. Adipocyte sizes were quantified from WAT histology slides and were quantified with Adiposoft software as described (20). Slides were imaged with microscope (Olympus BX-50, Olympus Optical, Japan).

### Immunohistochemistry

Adipose tissues were immediately excised and fixed in 4% paraformaldehyde after mice were sacrificed and then embedded in paraffin. Sections of 5 μm were blocked with 3% bovine serum albumin (BSA) and incubated overnight at 4°C with a monoclonal rabbit anti-F4/80 antibody (Servicebio, Wuhan, China) and afterward with a secondary antibody (Sangon Biotech, Shanghai, China) at room temperature for 1 h. The sections were then dehydrated in ethanol, cleared in xylene, and mounted with counterstaining followed by DAB staining (Servicebio, Wuhan, China). The slides were then photographed and converted into digital images with microscope (Olympus BX-50, Olympus Optical, Japan). Positive staining (dark brown) was quantified at × 400 magnification using Image J.

### Biochemical analyses

Blood samples were taken from the portal vein of mice. Plasma were separated by centrifugation at 10,000 rpm for 10 min at 4°C and stored at –80°C. Plasma glycerol and FFA concentrations were detected using kits from Nanjing Jiancheng Bioengineering Institute (Nanjing, China). Plasma insulin and IL-6 levels were measured by ELISA kits (Sangon Biotech, Shanghai, China). QUICK index [quantitative insulin sensitivity check index) was calculated using the formula: 1/(log (fasting insulin μU ml^–1^) + log (fasting glucose mg dl^–1^)]. Acetyl-CoA content in the liver was detected with an ELISA kit from Nanjing Jiancheng Bioengineering Institute (Nanjing, China).

### *In vitro* culture of adipose tissue explants

**FIGURE 1:**
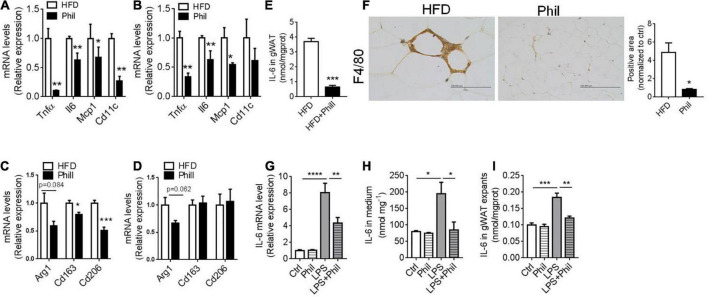
Phillyrin inhibits adipose tissue inflammation and IL-6 production. **(A–F)** HFD-fed mice treated with or without 25 mg per kg body weight Phillyrin. mRNA levels of M1 ATM in gWAT **(A)** and sWAT **(B)** in mice. *N* = 4 per group. mRNA levels of M2 ATM in gWAT **(C)** and sWAT **(D)** in mice. *N* = 4 per group. **(E)** IL-6 contents in gWAT in mice. *N* = 3 per group. **(F)** Representative images of IHC and quantification of F4/80 in gWAT in the groups indicated. Magnification × 400. Scale bars, 100 μm. *N* = 3 per group. **(G–I)** The gWAT pieces of WT mice were cultured for 24 h in DMEM containing 2% FFA-free BSA with 500 ng/ml LPS or 200 μM Phillyrin for 24 h. *N* = 3 per group. **(G)** mRNA levels of IL-6 in the gWAT explants. **(H)** IL-6 level in medium. *N* = 3 per group. **(I)** IL-6 content in the gWAT explants. All data in the figure were presented as mean ± SEM. *P < 0.05, **P < 0.01, ***P < 0.005, and ****P < 0.0001.

Following cervical dislocation, C57BL/6J mice were dissected and gWAT was isolated and cut into small pieces. The gWAT samples were then cultured in medium (DEMEM, 10% FBS, Invitrogen, USA) containing 1 mM sodium pyruvate, 2 mM glutamine. For the data used in Figures 1G–I, gWAT were treated with vehicle, 200 μM Phillyrin, 500 ng/ml LPS, or 200 μM Phillyrin + 500 ng/ml LPS for 24 h. Then, the medium was collected and the explants were washed 3 times with PBS and snap frozen for further assay. For basal lipolysis assay, around 20 mg gWAT samples were incubated in Hank’s balanced salt mixture (HBSS, Solarbio, China) containing 2% fatty-acid free BSA (Sangon Biotech, shanghai, China) at 37°C in indicated conditions. Glycerol concentrations in the medium were measured with a commercial kit from Nanjing Jiancheng Bioengineering Institute (Nanjing, China) and normalized to the total weight of the gWAT sample.

### Western blots

Tissues were homogenized in RIPA buffer supplemented with protease inhibitor, phosphatase inhibitor cocktail 1 and cocktail 2 (Servicebio, Wuhan, China), and protein were quantified using Bradford method (BIO-RAD). Proteins were electrophoresed on 10–15% SDS-polyacrylamide gels and transferred to 0.45 μm PVDF membranes (Millipore, Ireland) with a wet transfer system (BIO-RAD, USA) for 45 min. Membranes were blocked and incubated with primary antibodies in 3% BSA in Tris-Buffered Saline Tween-20 (TBST) overnight at 4°C. Blots were then rinsed and incubated with secondary antibodies conjugated to horseradish peroxidase (HRP) (Sangon Biotech, Shanghai, China) and exposed on an ECL chemiluminescence detection system (G&E Healthcare, USA). Information about sources of antibodies was listed as following: GAPDH (BOSTER, China), FAS (Santa Cruz, USA), ACC (Santa Cruz, USA), HSL (Cell signaling, USA), Phospho-HSL (Ser 563) (Affinity, China), PPARγ (Santa Cruz, USA), ATGL (Santa Cruz, USA), PLIN1 (BOSTER, China), and CGI-58 (Santa Cruz, USA).

### Quantitative real-time PCR

For the analysis of mRNAs, tissues were lysed and extracted with Trizol (Thermo Fisher Scientific, USA). 500 ng RNA was reverse-transcribed to cDNA using High-Capacity cDNA Reverse Transcription kit (Thermo Fisher Scientific, USA) following the manufacturer’s instructions. Gene expressions were measured using the FastStart Essential DNA Green Master kit (Roche, Germany). The amplification data were analyzed using Roche LightCycler 480 II (Roche, Germany). *Gapdh* was used as the housekeeping gene. Sequence of the primers was listed in Supplementary Table 1.

### RNA-sequencing analysis

Total RNA was extracted from gWAT explants *ex vivo* culture using Trizol according to the manufacturer’s instructions. The mRNA was then isolated with oligo magnetic beads and randomly fragmented using divalent cations in NEB fragmentation buffer for cDNA synthesis. Next-generation sequencing was performed on a BGISEQ-500 platform (BGI, China). After standard procedure to eliminate adapter contamination, low-quality reads, as well as excessive ambiguous bases by Trimmomatic software, Trinity was used to complete *de novo* assembly and the subsequent analysis regarding gene expression. Differentially expressed genes (DEG) were identified using DESeq2 based on negative binomial distribution (21). A *Q*-value (adjusted *P*-value) threshold of 0.05 and the ratio | Phil/Model| greater than 2 were chosen to create a volcano plot presenting the detected DEG. For visualization of the selected specific genes, pheatmap package from R project was used to plot the heatmap with default values.

### Data analysis

All values were presented as mean ± SEM. Statistical analyses were performed using PRISM 7.0 (GraphPad Software). Statistical significance was determined using Student’s *t*-test (two-tailed paired or unpaired) or by one-way ANOVA followed by the *post hoc* Dunnett’s and Turkey’s test. A *P*-value equal to or less than 0.05 was considered statistically significant. *P < 0.05, **P < 0.01, ***P < 0.005, and ****P < 0.0001.

## Results

### Phillyrin treatment protects mice from high-fat diet-induced obesity

**FIGURE 2:**
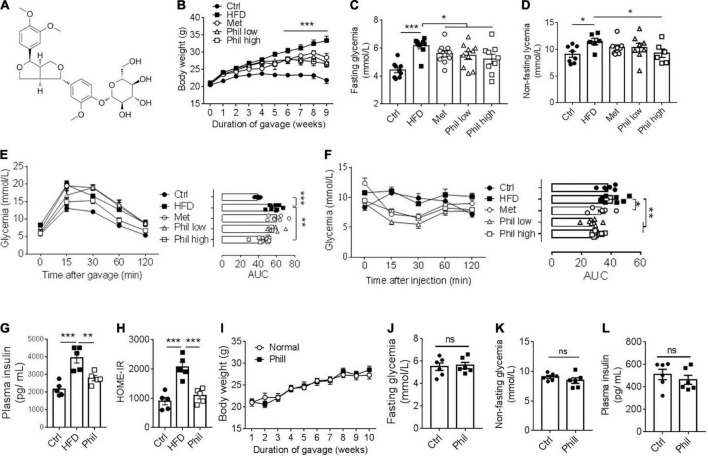
Phillyrin treatment improves obesity-related metabolic disorders. **(A)** Diagram of the chemical structure of Phillyrin. **(B)** Body weight of mice treated preventatively with Phillyrin at 25 mg and 50 mg per kg body weight, 1 mg per kg body weight metformin (Met) or vehicle control. *N* = 6–9 per group. Levels of fasting **(C)** and non-fasting **(D)** glycemia in mice. *N* = 6–9 per group. **(E)** Oral glucose tolerance test determined after 2 months of drug intervention by gavage of 2 g per kg body weight of glucose, and **(F)** insulin tolerance test in mice by i.p. injection of 0.05 U per kg body weight of insulin. *N* = 6–9 per group. Area under the curve (AUC) was calculated using GraphPad Prism software. **(G)** Plasma level of insulin and **(H)** HOME-IR index in HFD-fed mice treated with or without Phillyrin at 25 mg per kg body weight. *N* = 4–5 per group. **(I–L)** Four weeks old male C57BL/6J mice treated with or without Phillyrin (25 mg/kg) for 10 weeks. **(I)** Body weight of mice. *N* = 6 per group. Levels of fasting **(J)** and non-fasting **(K)** glycemia in mice. *N* = 6 per group. **(L)** Plasma level of insulin. *N* = 6 per group. All data in the figure are presented as the mean ± SEM. *P < 0.05, **P < 0.01, and ***P < 0.005. ns, not significant.

Phillyrin is one of the main active ingredients from *F. suspensa* (Figure 2A). Previous studies have indicated a potential role of Phillyrin in the regulation of energy homeostasis (19, 22). However, whether Phillyrin could retard obesity-associated metabolic disorders has not been thoroughly evaluated. To this end, wildtype (WT) C57BL/6J mice were exposed to HFD in the presence of different concentrations of Phillyrin for 9 weeks. Consistent to previous reports, we confirmed that Phillyrin treatment significantly prevented HFD-induced weight gain in mice as soon as from the fifth week after drug administration compared to HFD-fed control mice (Figure 2B). Moreover, Phillyrin treatment also downregulated the increased levels of fasting (Figure 2C) as well as non-fasting glycemia (Figure 2D) in HFD-fed mice compared to non-treated mice. Phillyrin also improved the impaired glucose clearance in HFD-fed mice in glucose (Figure 2E) and insulin tolerance tests (Figure 2F). Meanwhile, plasma insulin level was increased in HFD-fed mice in comparison to normal mice, which was normalized by Phillyrin treatment (Figure 2G). Consistently, Phillyrin treatment dramatically attenuated the augmented HOMA-IR index in mice fed by HFD compared with HFD-fed control mice (Figure 2H). Although HFD-fed mice ate 33% less than control animals, Phillyrin administration did not alter food intake in HFD-fed mice (Supplementary Figure 1), excluding the possibility that Phillyrin-associated metabolic benefits were due to hypophagia. Phillyrin administration did not alter body weight (Figure 2I), blood levels of glucose (Figures 2J,K) and insulin (Figure 2L) in chow-fed mice. Collectively, these findings suggest that Phillyrin treatment confers metabolic benefits in HFD-fed mice.

### Phillyrin treatment protects mice from high-fat diet-induced hepatic lipid accumulation

**FIGURE 3:**
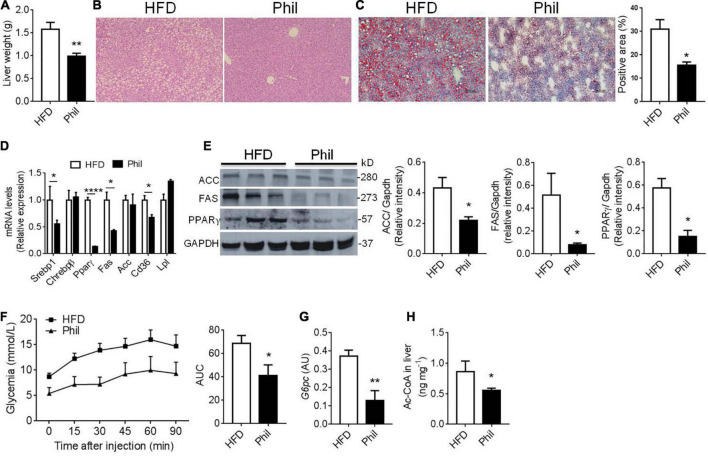
Phillyrin treatment alleviates hepatic lipid accumulation. **(A)** Total liver weight of HFD-fed mice gavaged with vehicle or with 25 mg per kg body weight Phillyrin. *N* = 4–5 per group. **(B)** Representative images of hematoxylin-eosin stained livers in the groups indicated. Magnification × 100. Scale bars, 100 μm. *N* = 3 per group. **(C)** Representative images of oil red O stained liver tissues (magnification × 100) and quantification of the staining in the groups indicated. *N* = 3 per group. **(D)** Expression of lipid metabolism related genes in the livers of mice in each group. *N* = 3–4 per group. **(E)** Protein levels of FAS, ACC, and PPARγ were detected in the livers. *N* = 3 per group. **(F)** Pyruvate tolerance test was determined after 2 months of drug intervention by i.p. injection of 2 g per kg body weight of pyruvate in mice in indicated groups. Area under the curve (AUC) was calculated using GraphPad Prism software. *N* = 5–6 per group. **(G)** mRNA level of *G6pc* in the livers of mice in each group. *N* = 4–5 per group. **(H)** Contents of acetyl-CoA in the liver in the groups indicated. *N* = 6 per group. All data in the figure are presented as mean ± SEM. *P < 0.05, **P < 0.01, and ****P < 0.0001.

We then further went to investigate Phillyrin’s influence on obesity-linked ectopic lipid deposition in the liver in HFD-fed mice treated preventatively with Phillyrin. The hepatomegaly normally observed in HFD-fed mice was markedly reversed by the treatment of Phillyrin, achieving around a 28% reduction in liver weight (Figure 3A). Moreover, results of hematoxylin-eosin (HE) and oil red O staining of the liver demonstrated that Phillyrin-treated mice exhibited significantly lower hepatic lipid contents compared to mice treated with vehicle control (Figures 3B,C). Concomitantly, Phillyrin-treated mice reduced the gene expression involved in lipid uptake, lipid storage and *de novo* lipogenesis (DNL) (*Srebp1* –45%, *Ppar*γ*1*–97%, *Fas* –58%, *Cd36* –33%, Figure 3D) in the liver compared to control mice. This result was confirmed by the protein levels of DNL pathway by western blot analysis (Figure 3E). Since increased HGP is a major contributor to elevated glycemia in obesity, pyruvate tolerance test (PTT) was assessed in mice. The result displayed that Phillyrin administration drastically reversed increased HGP under obesity (Figure 3F), associated with decreased expression of glycolytic gene *G6pc* in the liver (Figure 3G), the rate-limiting enzyme driving gluconeogenesis in HFD-fed mice. In addition, hepatic acetyl-CoA content was also reduced by Phillyrin treatment in HFD-fed mice relative to non-treated control mice (Figure 3H). With regard to the essential role of hepatic acetyl-CoA in the mediation of WAT-derived FFA in hepatic insulin insensitivity (2), we hypothesized that Phillyrin treatment could reduce the FFA flux from dysfunctional WAT to liver in obesity.

### Phillyrin reduces IL-6 production in white adipose tissue

Next, we continued to investigate the possible effects of Phillyrin on WAT during obesity. Concomitant to weight loss, accumulation of fat mass were reduced in Phillyrin-treated animals compared to HFD-fed counterparts (Supplementary Figure 2A). Increases in size of adipocytes (hypertrophy) promote adipose tissue inflammation and insulin resistance in obesity (23). To determine the dynamic change of adipocytes, HE staining (Supplementary Figure 2B) was performed and the quantification of adipocyte size showed that average adipocyte diameters were markedly reduced in gWAT of Phillyrin-treated mice vs. HFD-fed control mice (Supplementary Figure 2C). To evaluate whether Phillyrin antagonized obesity-associated inflammation in fat tissue, quantitative real-time PCR (qPCR) analysis of RNA samples of perigonadal (g) WAT and subcutaneous (s)WAT from HFD-fed mice treated with or without Phillyrin was performed. The result showed that Phillyrin treatment dramatically reduced messenger RNA (mRNA) levels of characteristic M1 ATM markers in gWAT including *Tnf*α (–89%), *Il-6* (–36%), *Mcp1* (–32%), and *Cd11c* (–72%) as compared to HFD-fed control mice (Figure 1A). In sWAT, levels of *Tnf*α (–54%) and *Il-6* (–81%) were also drastically declined in Phillyrin-treated mice (Figure 1B). However, Phillyrin treatment also slightly decreased the M2 ATM markers in gWAT including *Cd163* (–21%) and *Cd206* (–50%) in mice compared to HFD-fed control mice (Figure 1C). There was also a great trend for *Arg1* (*P* = 0.062) to be reduced by Phillyrin treatment in sWAT in mice compared with HFD-fed mice (Figure 1D). Based on the findings of Perry et al. the elevation of IL-6 is more sensitive than any other inflammatory mediators in plasma and adipose tissue macrophages in HFD-fed mice (2), as well as emerging evidence that IL-6 is an important mediator under adipose tissue inflammation-related lipolysis in obesity (24–27), we then focused on the effect of Phillyrin treatment on IL-6 in WAT. Additionally, IL-6 content in gWAT in HFD-fed mice was dramatically decreased by Phillyrin treatment compared to non-treated mice (Figure 1E). Finally, the result of immunohistochemistry (IHC) confirmed that formation of crown-like structure (CLS) in gWAT in HFD-fed mice was markedly inhibited by Phillyrin treatment (Figure 1F). To gain a better view of the role of Phillyrin in adipose inflammation, we examined the effects of Phillyrin *in vitro* in the gWAT explants stimulated with lipopolysaccharide (LPS). As a result, LPS stimulation dramatically elevated the mRNA level of *IL-6* which was restored by treatment of Phillyrin (Figure 1G). Moreover, Phillyrin treatment in the gWAT explants in the presence of LPS successfully reduced IL-6 secretion (Figure 1H) and its cellular content (Figure 1I). Phillyrin treatment alone did not affect IL-6 expression or secretion (Figures 1G–I). In summary, these data indicate that Phillyrin treatment alters the ATM expression profile in HFD-fed mice and inhibits IL-6 production in WAT.

### Phillyrin antagonizes IL-6-induced basal lipolysis with adipose triglyceride lipase inhibition *in vitro*

**FIGURE 4:**
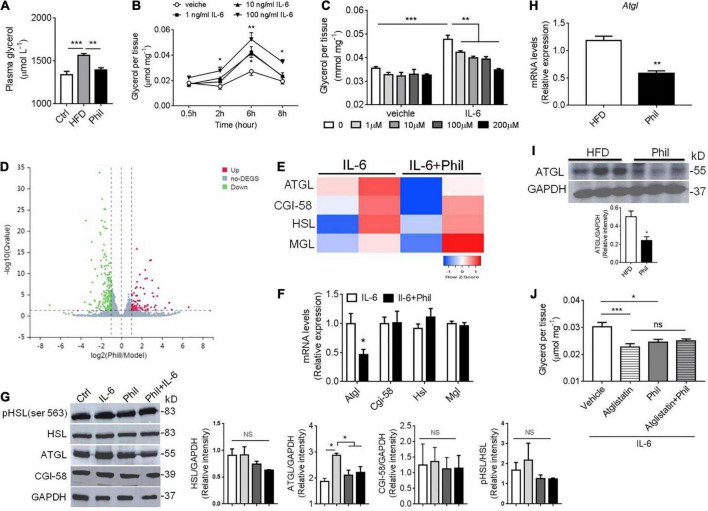
Phillyrin decreases IL-6-related basal lipolysis associated with ATGL inhibition. **(A)** Plasma level of glycerol in mice fed a normal diet or HFD in the presence or absence of Phillyrin (25 mg per kg body weight). *N* = 4–5 per group. **(B)** Time-dependent alterations of basal lipolysis in the gWAT explants of WT mice in the presence of 0, 1, 10, 100 ng/ml IL-6. *N* = 3 per group. **(C)** Effects of Phillyrin ranged from 0, 1, 10, 100, 200 μM on basal lipolysis in the presence of 100 ng/ml IL-6 or not were assessed *in vitro* in the gWAT pieces of WT mice cultured for 6 h. *N* = 4 per group. **(D)** A volcano plot of gene expression elaborating the up-regulated and down-regulated genes with a total number of 680 represented by claret and green dots, respectively **(E)** a heatmap of genes involved in lipolysis in the gWAT explants treated with or without 200 μM Phillyrin in the presence of 100 ng/ml IL-6 for 24 h. *N* = 2 per group. **(F)** mRNA levels of *ATGL, CGI-58, HSL, and MGL* in the gWAT explants in the gWAT explants treated with or without 200 μM Phillyrin in the presence of 100 ng/ml IL-6 for 24 h. *N* = 6 per group. **(G)** Representative images of immunoblots of lysates of lipolytic proteins from control (Ctrl), 100 ng/ml IL-6 stimulated gWAT explants or Phillyrin-treated gWAT explants in the presence of IL-6 for 24 h. *N* = 3 per group. **(H)** mRNA level of ATGL in HFD-fed mice treated with or without Phillyrin (25 mg per kg body weight). *N* = 4 per group. **(I)** Protein level of ATGL in HFD-fed mice treated with or without Phillyrin (25 mg per kg body weight). *N* = 3 per group. **(J)** Basal lipolysis in the gWAT pieces of WAT mice treated with vehicle, atglistatin (25 μM), Phillyrin 200 μM or atglistain + Phillyrin in the presence of 100 ng/ml IL-6 for 6 h. *N* = 6 per group. All data in the figure were presented as mean ± SEM. *P < 0.05, **P < 0.01, and ***P < 0.005.

Since IL-6 accounts significantly for obesity-related basal lipolysis in adipose tissue (2), we went on to test whether Phillyrin treatment could lower plasma level of glycerol, a product of lipolysis, in HFD-fed mice. Indeed, the result showed that the elevated plasma level of glycerol was markedly decreased by Phillyrin treatment in HFD-fed mice (Figure 4A), supporting that Phillyrin would possibly restore unrestrained basal lipolysis under obesity. We then modeled inflammation-related lipolysis by culturing the gWAT with IL-6 *in vitro*. The result showed that IL-6 at concentrations ranging from 1 to 100 ng/ml all could significantly drive the basal lipolysis in gWAT explants in a time-dependent manner (Figure 4B). Next, we went on to investigate whether different concentrations of Phillyrin would interfere with basal lipolysis in the presence of IL-6 or not. Notably, while Phillyrin treatment alone did not dramatically affect basal lipolysis in adipose tissue, Phillyrin at concentrations ranging from 1 to 200 μM all could significantly reduce IL-6-linked basal lipolysis in gWAT explants *in vitro*, with 200 μM completely blocking IL-6-induced increase in basal lipolysis (Figure 4C). To obtain a detailed transcriptomic profile regarding the effect of Phillyrin on lipolysis, we performed RNA sequencing (RNA-seq) on the gWAT explants treated with or without Phillyrin in the presence of IL-6 *in vitro*. Notably, Phillyrin treatment resulted in 680 DEG with *P*_*adjusted*_ < 0.05 and | log2 fold change| > 1 (Figure 4D). Among them, the expression of lipolytic gene *ATGL* was dramatically reduced by Phillyrin treatment while *HSL*, *MGL*, or *CGI-58* were not altered in IL-6-stimulated gWAT explants (Figure 4E). These results were then further validated by qPCR analysis in which only the *ATGL* mRNA level was suppressed by Phillyrin treatment in IL-6-exposed gWAT explants (Figure 4F). Concomitantly, Phillyrin treatment reversed the elevated ATGL protein level stimulated by IL-6 in the gWAT explants. Phosphorylation of HSL at Ser 563 a responsive site to protein kinase A (PKA) activation, and CGI-58 were not altered by IL-6 or Phillyrin treatment in the gWAT explants. Phillyrin treatment alone did not change the protein level of ATGL in normal gWAT explants *in vitro* (Figure 4G).

Thus, these results suggest that Phillyrin could abrogate IL-6-related basal lipolysis and lipid breakdown in adipose tissue via negatively regulating expression of ATGL. We then went back to see if these results would be retracted in obese model. Indeed, both the mRNA and protein levels of ATGL were decreased in adipose tissue in the HFD-fed mice treated with Phillyrin (Figures 4H,I). Moreover, Phillyrin treatment would not further reduce IL-6-driven basal lipolysis in gWAT, if ATGL was already inhibited by the ATGL inhibitor Atglistatin (Figure 4J). Phillyrin treatment did not affect plasma level of glycerol or lipolytic protein expression in chow-fed mice (Supplementary Figure 3). Thus, these data led us to hypothesize that Phillyrin potentially exhibits an anti-lipolytic effect against IL-6-related lipolysis through inhibiting ATGL expression in WAT.

### Phillyrin reverses IL-6-associated basal lipolysis and hepatic dysfunctions *in vivo*

**FIGURE 5:**
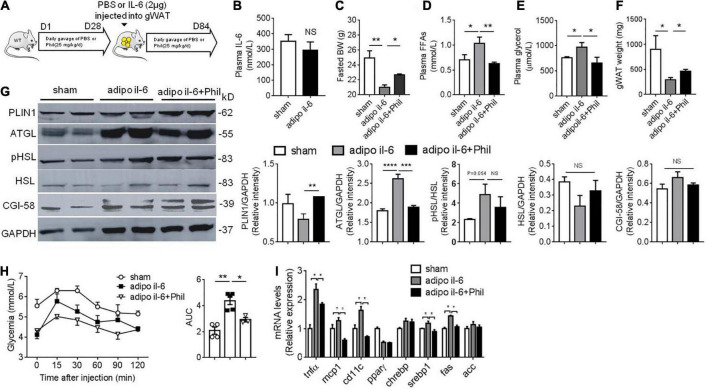
Phillyrin attenuates adipose IL-6-associated basal lipolysis and hepatic dysfunctions. **(A)** Protocol of IL-6 injection into WAT *in vivo*. WT mice previously treated with or without Phillyrin (i.g. 25 mg/kg/d) for 28 days, received IL-6 injection into gWAT (1 μg per depot per mouse) and after that Phillyrin treatment continued for another 56 days. Sham mice also surgically received PBS injection into gWAT. **(B)** Plasma level of IL-6 of sham and adipo il-6 mice. *N* = 6 per group. **(C)** Body weight of sham mice or adipo IL-6 mice treated with vehicle or Phillyrin. *N* = 4–7 per group. **(D)** Plasma FFA level, **(E)** plasma glycerol level, and **(F)** total gWAT weight in mice. *N* = 4–7 per group. **(G)** Western blot analysis of lipolytic proteins expression and HSL phosphorylation at Ser 563 in gWAT of mice. *N* = 4 per group. **(H)** Pyruvate tolerance test and AUC of different groups. *N* = 4–7 per group. **(I)** Expression of inflammation and lipid metabolism related genes in the livers of mice. *N* = 3–4 per group. All data in the figures are shown as mean ± SEM. *P < 0.05, **P < 0.01, ***P < 0.005, and ****P < 0.0001.

To further confirm the result that Phillyrin reduces augmented basal lipolysis associated with IL-6 and subsequent hepatic dysfunctions *in vivo*, WT mice previously treated with or without Phillyrin were surgically received one shot of IL-6 injection directly into depots of gWAT (Figure 5A). Plasma level of IL-6 was not changed by adipose IL-6 injection (Figure 5B), limiting the effects of IL-6 in gWAT in mice. In line with expectation, adipose IL-6 injection initiated a dramatic loss of body weight by nearly 4-g in mice compared to sham mice, which was reversed up to 1.7-g by Phillyrin treatment (Figure 5C). The elevated plasma levels of FFA (Figure 5D) and glycerol (Figure 5E) in mice with adipose IL-6 injection indicated promotion of basal lipolysis and lipid breakdown in adipocytes compared with sham mice. In consistent, Phillyrin treatment suppressed the increased plasma FFA (Figure 5D) and glycerol (Figure 5E) levels by adipose IL-6 injection. In addition, the marked loss of fat weight might account for total weight waste induced by adipose IL-6 injection in mice, which was preventable by Phillyrin supplementation (Figure 5F). Remarkably, western blot studies confirmed elevated expression of ATGL in gWAT in mice received adipose IL-6 injection, which was abrogated by Phillyrin treatment. Although PLIN1 protein level was not significantly altered by adipose IL-6 injection, Phillyrin profoundly elevated its level in gWAT in mice compared to non-treated mice injected with IL-6. Phillyrin exhibited no alteration in phospho-HSL, HSL and CGI-58 protein levels (Figure 5G). Therefore, these data suggest that Phillyrin could block IL-6-stimulated basal lipolysis *via* decreasing ATGL protein level in adipose tissue *in vivo*. Although there was no alterations in glucose tolerance (data not shown), PTT experiment showed that adipose IL-6 injection significantly elevated HGP which was reduced by Phillyrin treatment in mice (Figure 5H). We also measured the expression of key metabolic and inflammatory genes in the livers to investigate the hepasteatic phenotype. In line, Phillyrin treatment reversed the increased gene expressions involved in inflammation including *Tnf*α, *Mcp1* and *Cd11c* in the liver in mice with adipose IL-6 injection (Figure 5I). The liver of adipo il-6 mice was also associated with higher expressions of *Srebp1* and *Fas*, which were lowered by Phillyrin treatment (Figure 5I). Therefore, these data further confirm that Phillyrin treatment attenuates IL-6-associated basal lipolysis and subsequent lipid spillover from adipose tissue by suppressing ATGL expression.

## Discussion

High circulating FFA level is a well-established risk factor for the development of obesity-associated hepatic steatosis (10, 28). Mounting evidence has suggested a role of adipose tissue-liver crosstalk in obesity-induced hepatic steatosis (29–31). Amongst, adipose tissue inflammation is recognized as an important factor contributing to accelerated basal lipolysis from adipocytes and subsequent lipid spillover into liver during obesity (2, 12, 13, 32). Consequently, we hypothesized that pharmacological compounds that have anti-inflammatory property could attenuate obesity-associated ectopic lipid accumulation by relieving adipose tissue inflammation. Our current study investigated this possibility with Phillyrin which is frequently prescribed in the clinic for treatments of respiratory infections in China (33). We demonstrated that HFD-fed mice treated with Phillyrin chronically remained more insulin sensitive and resistant to hepatic lipid disposition or HGP elevation. Meanwhile, Phillyrin treatment resulted in diminished lipid breakdown from adipose tissue, associated with reduced adipose tissue inflammation and IL-6 production in obese mice. Furthermore, we demonstrated that Phillyrin possessed an anti-lipolytic property against IL-6-induced basal lipolysis in adipose tissue both *in vivo* and *in vitro* by negatively targeting ATGL. Finally, Phillyrin may represent a promising chemical compound in the field of obesity, obesity-related adipose tissue inflammation and hepatic steatosis.

*F. suspensa* has long been used for respiratory infectious diseases in China and Phillyrin could decrease plasma levels of various cytokines in inflamed states. Herein, we showed that Phillyrin treatment could also decrease adipose tissue inflammation in HFD-fed mice, indicated by reductions in proinflammatory gene markers of both M1 and M2 macrophages. To explore whether Phillyrin could directly target adipose tissue or the lowered inflammation in this organ in obesity is secondary to its systemic effects, we then modeled inflammation in adipose tissue explants *in vitro* with LPS treatment, which suggested that this compound could directly relieve adipose tissue inflammation induced by LPS. Meanwhile, this compound substantially reduced HFD-induced hepatic lipid accumulation and HGP, as well as the expression of several pro-steatotic genes including *Cd36*, *Fas*, *Acc*, *Ppar*γ and *Screbp1* in HFD-fed mice. We then wondered whether the adipose-liver crosstalk had a role in the benefited phenotype of liver in Phillyrin-treated obese mice. In light by the work of Perry et al. (2), who have revealed that hepatic acetyl-CoA links adipose tissue inflammation to hepatic HGP and lipid accumulation, we went on to check if Phillyrin treatment would alter the hepatic level of acetyl-CoA. Indeed, the result showed that Phillyrin-treated obese mice had less acetyl-CoA in the liver. Therefore, we hypothesized that Phillyrin treatment could restrain FFA transport from inflamed adipose tissue to liver in obesity.

Recently, the role of IL-6 in connecting inflammation and lipid breakdown in adipose tissue is gaining importance (14, 34–36). Blocking IL-6 *trans*-signaling prevents HFD-induced metabolic disorders in mice (37). Thus, adipose IL-6 might be a potential target for correcting obesity-related complications. Our results demonstrated that IL-6 level in gWAT was dramatically reduced by Phillyrin treatment in HFD-fed mice. Thus, in our current work we then focused on the role of Phillyrin treatment on IL-6-linked basal lipolysis in adipose tissue. We firstly modeled inflammation-linked basal lipolysis in adipose tissue by treating gWAT explants with IL-6 *in vitro*. Indeed, our result demonstrated that IL-6 at concentrations ranging from 1 to 100 ng/ml all could significantly elevate basal lipolysis in adipose tissue explants in a time-dependent manner. Interestingly, treatment of Phillyrin resulted in resistance to the increase in basal lipolysis induced by IL-6 in gWAT *in vitro*.

Despite the well-known role of IL-6 in basal lipolysis, the underlying molecular mechanisms are not yet fully understood. Herein, we demonstrated that increased ATGL protein level possibly accounted for IL-6-related basal lipolysis in adipose tissue, with other lipolytic proteins including CGI-58, HSL, and phosphor-HSL (ser 563) not altered. Intriguingly, IL-6 did not increase the transcriptional level of ATGL in gWAT explants *in vitro* (Supplementary Figure 4), indicating post-transcriptional regulation of ATGL by IL-6, which merits further investigation. Nevertheless, the anti-basal lipolytic effect of Phillyrin treatment against IL-6 was linked with reduced expression of ATGL at both protein and mRNA levels. According to Hong et al., activation of ERK1/2 signaling contributes to upregulated basal lipolysis by cytokines in adipose tissue (38). Meanwhile, it is also reported that Phillyrin could inhibit LPS-induced ERK1/2 signaling in bone marrow-derived macrophages *in vitro* (15). We then studied whether ERK1/2 signaling participate in the inhibition of IL-6-linked basal lipolysis by Phillyrin. In fact, the results did show that Phillyrin treatment could abrogate the phosphorylation and activation of ERK1/2 induced by IL-6 in gWAT explants (Supplementary Figure 5). However, we did not observe reduction in basal lipolysis induced by IL-6 with the treatment of ERK1/2 inhibitor U0126 in gWAT explants (Supplementary Figure 6). Thus, these data excluded the contribution of ERK1/2 inhibition to the anti-lipolytic effect of Phillyrin against IL-6. Further studies are needed to elucidate how exactly Phillyrin treatment can downregulate IL-6-associated basal lipolysis and ATGL activation.

To further validate that Phillyrin treatment could blunt IL-6-linked basal lipolysis and ectopic lipid spillover *in vivo*, we then created a mouse model of IL-6 injection into gWAT (adipo il-6 mice), where adipo il-6 mice demonstrated weight loss accounted mostly by reduced fat mass, which were reversed with Phillyrin treatment. The loss of fat mass in adipo il-6 mice was associated with increased plasma levels of FFA and glycerol, and increased expression of ATGL in gWAT in mice, indicating elevated basal lipolysis. Concomitantly, all of these changes in adipo il-6 mice were attenuated by Phillyrin treatment. Furthermore, the result of PTT showed that fat IL-6 injection significantly increased HGP in mice compared with sham mice, which was also reduced by Phillyrin treatment. Likewise, Phillyrin treatment ameliorated the upregulated inflammation and lipogenesis in adipo il-6 mice.

Our findings implicate Phillyrin treatment could correct diet-induced obesity, glucose intolerance, adipose tissue inflammation and fatty liver in mice, representing a promising candidate for the treatment of obesity and related hepatic steatosis. Specifically, Phillyrin is a promising compound that can completely reverse IL-6-linked basal lipolysis and lipid spillover from adipose tissue associated with ATGL inhibition. Thorough mechanistic studies are warranted to further illuminate how Phillyrin regulates ATGL expression in the presence of IL-6 in adipose tissue.

## Data availability statement

The original contributions presented in this study are included in the article/Supplementary material, further inquiries can be directed to the corresponding author/s.

## Ethics statement

The animal study was reviewed and approved by the Institutional Animal Care and Use Committee (IACUC) at Anhui University of Chinese Medicine.

## Author contributions

XT designed the experiments, wrote the manuscript draft, and supervised the experimentators. RH corrected the draft. ZF, LW, YL, and HH performed the experiments. TW and RH participated in the discussion. All authors agreed to be accountable for all aspects of work ensuring integrity and accuracy.
