# Supplementary material for: Phillyrin restores metabolic disorders in mice fed with high-fat diet through inhibition of interleukin-6-mediated basal lipolysis
Source: Front Nutr. 2022 Oct 5;9:956218. doi: 10.3389/fnut.2022.956218 (PMC9581271; doi:10.3389/fnut.2022.956218)
Supplement: Supplementary file 1 [file Table_1.DOCX]

Supplementary Material

# Supplementary Figures and Tables

**1.1 Supplementary Figures**

**Supplementary Figure 1.** Effects of Phillyrin on food intake in HFD-fed mice. Four weeks old male C57BL/6J mice were fed HFD in the presence or absence of Phillyrin (Phil) for nine weeks. All data in the figure were presented as mean ± SEM. **P*<0.05.

**Supplementary Figure 2.** Effects of Phillyrin on adipose tissue in HFD-fed mice. HFD-fed mice treated with or without 25 mg per kg body weight Phillyrin. (A) Tissue weight of gWAT and sWAT in mice. N=4-5 per group. (B) Representative images of HE stained gWAT in the groups indicated. Magnification × 100. Scale bars, 100 μm. N=3 per group. (C) Quantification of adipocyte size distribution of gWAT in mice. N=3 per group. All data in the figure were presented as mean ± SEM. **P*<0.05, ***P*<0.01, *****P*<0.01.

**Supplementary Figure 3.** Plasma level of glycerol (A) and immunoblots of liplyitc proteins in perigonadal adipose tissues (B) in chow-fed C57BL/6J mice treated with or without Phillyrin (25 mg/kg).

**Supplementary Figure 4.** Effects of IL-6 on mRNA level of ATGL in the gWAT explants. N=3 per group. All data in the figure were presented as mean ± SEM.

**Supplementary Figure 5**. Representative images of immunoblots of lysates of pERK1/2 and ERK1/2 in the gWAT explants treated with 100 ng/ml IL-6 or 200 μM Phillyrin.

**Supplementary Figure 6.** Basal lipolysis in the gWAT pieces of WAT mice treated with vehicle or U0126 (20 μM) in the presence of 100 ng/ml IL-6 for 6h. N=6 per group. All data in the figure were presented as mean ± SEM. ns, not significant.

# 1.2 Supplementary Table

**Supplementary Table 1. Primer Sequences for qPCR Gene Expression Analysis.**

| **Gene** | **Forward Primer (5’->3’)** | **Reverse Primer (5’->3’)** |
| --- | --- | --- |
| Srebp1 | CTGGCTTGGTGATGCTATGTTG | GACCATCAAGGCCCCTCAA |
| Chrebpbeta | ACATCAGCGCTTTGACCAGAT | TGCGCGTCCGGACATAG |
| Pparr | CTGTTTTATGCTGTTATGGGTGAAA | GCACCATGCTCTGGGTCAA |
| Fas | ATCCTGGAACGAGAACACGATCT | AGAGACGTGTCACTCCTGGACTT |
| Acc | CTCCTTTGCCTTCCGACATC | TACCATGCCAATCTCATTTCCTC |
| Cd36 | GGACATACTTAGATGTGGAACCCATA | TGTTGACCTGCAGTCGTTTTG |
| Lpl | TTATCCCAATGGAGGCACTTTC | CACGTCTCCGAGTCCTCTCTCT |
| G6pc | ACGTATGGATTCCGGTGTTTG | CAGCTGCACAGCCCAGAA |
| Il-6 | GCCCACCAAGAACGATAGTCA | CAAGAAGGCAACTGGATGGAA |
| Tnfa | TGGGACAGTGACCTGGACTGT | TTCGGAAAGCCCATTTGAGT |
| Angptl4 | GTTTGCAGACTCAGCTCAAGG | CCAAGAGGTCTATCTGGCTCTG |
| Dgat2 | AGCTGGTGAAGACACACAACC | TGATGATAGCATTGCCACTCC |
| Mcp1 | GCAGTTAACGCCCCACTCA | CCAGCCTACTCATTGGGATCA |
| Cd11c | GATTTCAGCATCCCAGATCCC | CCAGATCCACCAGTCCATCC |
| Atgl | CGCCTCTCGAAGGCTCTCTT | TGTAGCCCTGTTTGCACATCTC |
| Cgi-58 | GAGAACCCAAAGGAGAGTTGCT | GACCTTGCTCCTTATTGAAGCC |
| Hsl | GACCATCAACCGACCAGGA | AGCAGCCTTTGTGTAGCGTG |
| Mgl | GGATGGGACCGACTTTGAGAA | TCCGTGTCCGAACCAGTTATCT |
| Gapdh | TGCACCACCAACTGCTTAGC | GGCATGGACTGTGGTCATGAG |
