# Supplementary figures and images for: Phillyrin restores metabolic disorders in mice fed with high-fat diet through inhibition of interleukin-6-mediated basal lipolysis
Source: Front Nutr. 2022 Oct 5;9:956218. doi: 10.3389/fnut.2022.956218 (PMC9581271; doi:10.3389/fnut.2022.956218)

Fig.S1

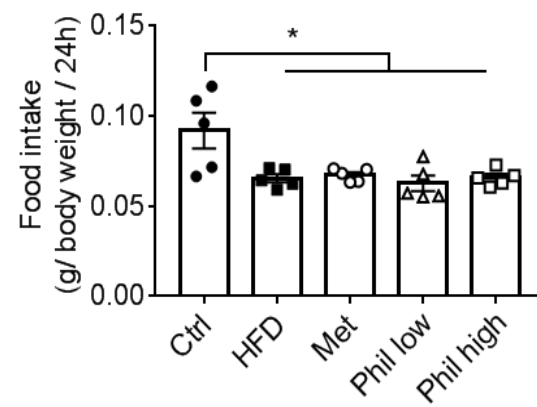

Fig.S2

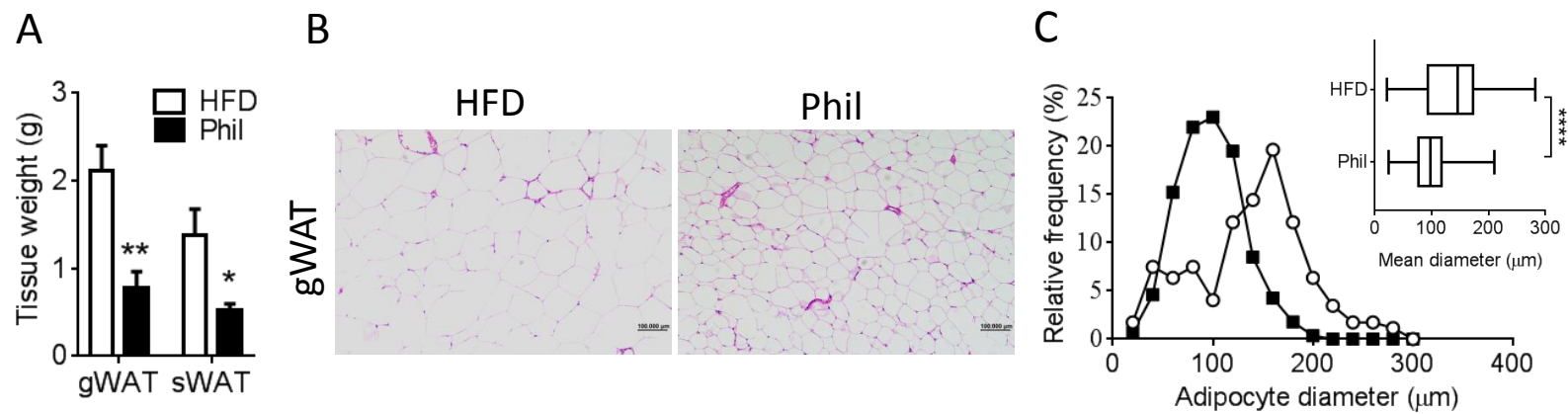

Fig.S3

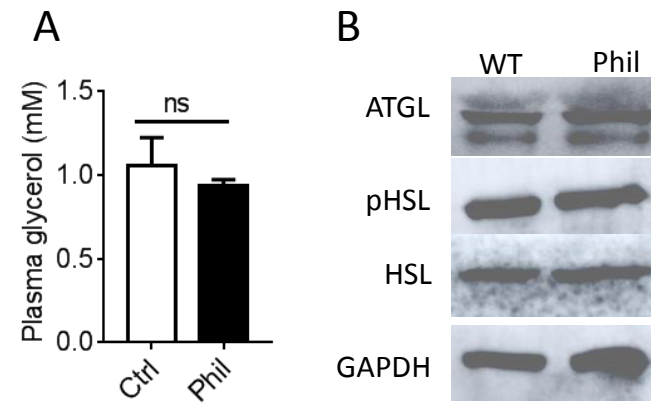

Fig.S4

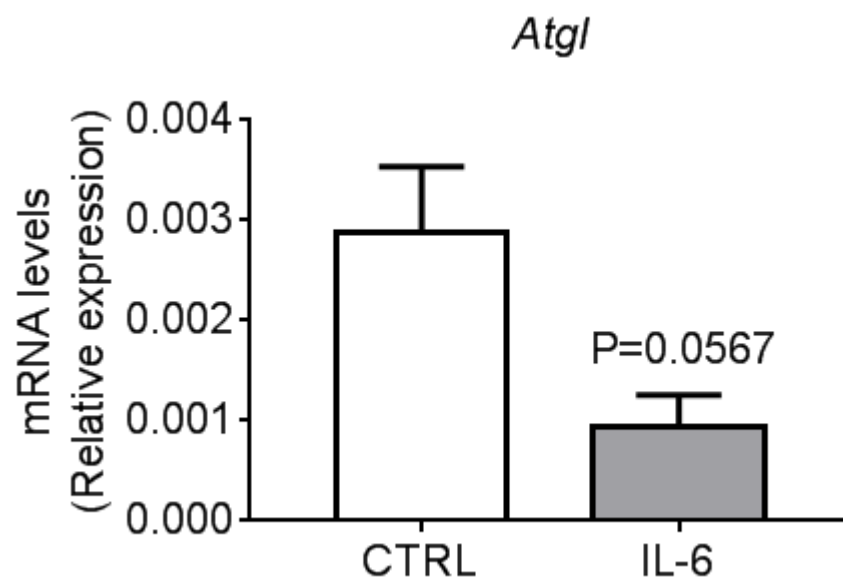

Fig.S5

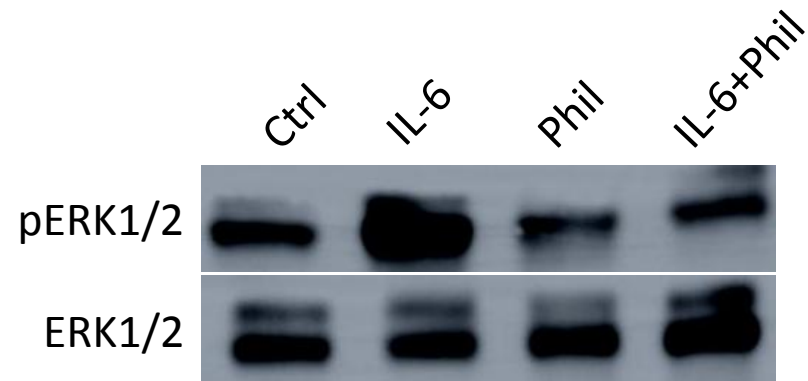

Fig.S6

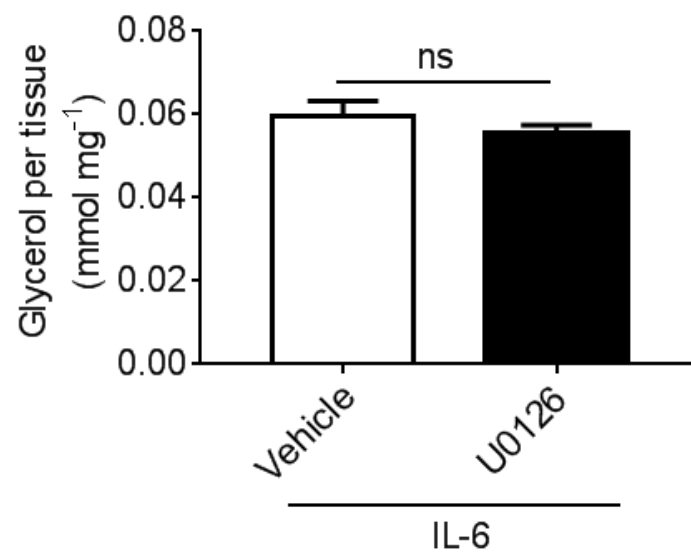

Supplement: Supplementary file 2 [file Data_Sheet_1.PDF]
